# Intelligent Support for Radiotherapy: A Review of Clinical Applications for Large Language Models

**DOI:** 10.3390/jcm15072531

**Published:** 2026-03-26

**Authors:** Juanjuan Fu, Yifan Cheng, Zhaobin Li, Jie Fu

**Affiliations:** 1 Department of Radiation Oncology, Shanghai Sixth People’s Hospital Affiliated to Shanghai Jiao Tong University School of Medicine, Shanghai 200233, China; 15951993619@163.com (J.F.); lizhaobin79@163.com (Z.L.); 2 Department of Radiation Oncology, Shanghai Chest Hospital, Shanghai Jiao Tong University, Shanghai 200233, China; 13701679825@163.com

**Keywords:** large language models, radiotherapy, treatment planning, auto-delineation, radiation therapy, clinical decision support

## Abstract

**Background**: Radiotherapy (RT) is a core modality for cancer treatment, yet it is plagued by inter-observer variability in target delineation, inefficient manual workflows, and challenges in fusing multi-type clinical data. Large language models (LLMs), with their superior semantic understanding and cross-modal fusion capabilities present novel solutions to these challenges. **Scope**: This narrative review provided a comprehensive overview of the current landscape and emerging trends of LLM applications across the entire RT workflow. **Findings**: LLMs demonstrated substantial clinical utility in key RT domains, including automated target volume delineation (e.g., Medformer, Radformer), dose prediction (e.g., DoseGNN), treatment planning automation (e.g., GPT-Plan), patient education, clinical decision support, medical information extraction, and prognosis assessment. These applications not only have the potential to enhance the accuracy and efficiency of RT but also facilitate the standardization of clinical pathways. However, widespread clinical adoption was impeded by critical limitations, including model hallucinations, insufficient generalizability, and unresolved issues regarding data privacy and ethical governance. **Conclusions**: LLMs possessed transformative potential to revolutionize radiation oncology. Future endeavors should prioritize technical refinements to mitigate model deficiencies, establish standardized evaluation benchmarks, and develop robust ethical frameworks. These concerted efforts are crucial for translating LLM research into clinical practice and advancing the era of intelligent, precision RT.

## 1. Introduction

Radiotherapy (RT) is a commonly employed modality in cancer treatment, serving as a localized therapeutic approach that utilizes high-energy radiation to precisely destroy tumor cells [1]. RT is widely used to treat various types of cancer, including lung, cervical, prostate, and head and neck cancers. The efficacy of RT is intrinsically linked to the precision of the treatment plan, the accuracy of target volume delineation, and the standardization of the treatment workflow [2,3]. The RT workflow involves several stages, including patient positioning for simulation, delineation of the target volume and organs at risk (OARs), treatment plan optimization, dose verification, and treatment delivery. Formulating the treatment plan requires physicists to repeatedly adjust parameters in order to balance achieving an optimal radiation dose to the planned target volume (PTV) with minimizing exposure to the surrounding OARs. This constitutes a complex optimization problem involving multiple conflicting objectives [4,5]. Target volume delineation relies on the clinical experience and subjective judgement of radiation oncologists [6], making it susceptible to variations in clinical practices and differences in clinical protocols across treatment centers. Consequently, inter- and intra-observer variability is unavoidable, significantly increasing the clinical workload while potentially compromising the consistency and reliability of treatment plans [7,8].

Significant progress has been made in the application of artificial intelligence (AI) technologies to the field of radiation oncology. Deep learning models have demonstrated certain advantages in organ segmentation and dose prediction, among other areas; nevertheless, several limitations still exist, including reliance on extensive annotated datasets, limited generalization capabilities, and difficulties in integrating clinical textual information [9,10,11]. In recent years, large language models (LLMs) such as ChatGPT, Llama 3.2, and Qwen-2.5 have rapidly emerged. Through pretraining on vast text datasets, these models possess exceptional semantic comprehension, logical reasoning, contextual learning capabilities, and cross-modal fusion advantages, offering novel approaches to addressing complex challenges in RT [8].

Existing reviews of the application of AI in RT primarily focus on deep learning algorithms for specific clinical tasks. They lack analysis of the integration of LLMs throughout the entire RT process, as well as their clinical safety and adaptability. This study, therefore, adopted a narrative review methodology based on the latest research findings to analyze the value of LLMs in enhancing treatment efficiency and quality, as well as optimizing clinical workflows. It also explored the limitations of current technologies and future development trends. This review aims to provide theoretical support and practical guidance for the standardized and personalized application of LLMs in RT.

**Figure 1:**
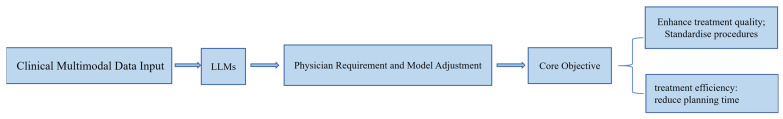
Full-process application framework of LLMs in RT.

As shown in Figure 1, the framework clearly presented a simplified clinical workflow of LLMs in the field of RT, aiming to intuitively demonstrate their application potential and practical value in the entire workflow of RT. The workflow makes full use of the powerful natural language processing and multimodal information fusion capabilities of LLMs to efficiently analyze and deeply mine clinically relevant data. Meanwhile, targeted model optimization and iteration were performed according to the actual clinical needs of radiation therapists so as to better adapt to the clinical scenarios of RT and further improve the practicability and reliability of the model.

This review categorized LLM-related models into three types based on their structural composition and functional core, which provides a unified analytical framework for the subsequent critical comparison of LLM applications in different RT fields and avoids conceptual ambiguity.

LLM-based model: Standalone models that use LLMs as the sole core architecture, relying only on natural language processing capabilities to complete clinical tasks, without integrating any imaging processing or domain-specific non-text algorithms. Typical use cases include purely text-based tasks, such as medical terminology standardization, patient education, and extraction of unstructured clinical text information. LLM-assisted model: Hybrid workflow architecture where the LLM serves only as a decision-making and guidance module. Its core function is to extract rules from clinical guidelines and literature, generate task-specific prompts, and optimize workflow logic. The main clinical tasks are performed by specialized non-LLM algorithms. Multimodal foundation model: Extended architectures based on LLMs, integrating visual/cross-modal understanding capabilities, capable of processing textual clinical information (guidelines, reports, prescriptions) and medical imaging data (CT/MRI/PET-CT) together, achieving multisource data fusion. They are specifically designed to address complex clinical tasks in RT that require multimodal data support, such as automatic target delineation and comprehensive treatment plan optimization.

**Figure 2:**
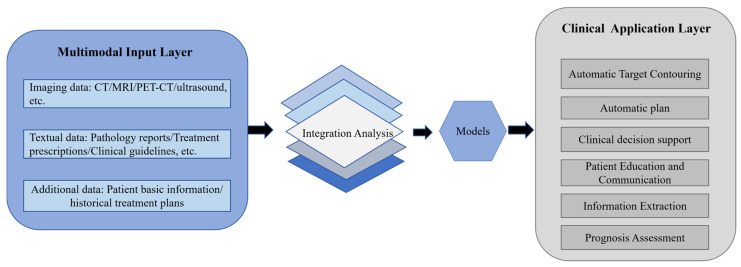
A multimodal foundation LLM framework for RT applications. The left panel shows the three main types of clinical multimodal data: imaging data, text data and supplementary data reflecting patient-specific characteristics. The right panel outlines six core applications for LLMs, these applications covered critical stages in RT, including target delineation, plan design, decision support, communication and prognosis prediction, fully demonstrating the comprehensive application potential of LLMs throughout the entire clinical RT workflow. Note: computed tomography (CT); magnetic resonance imaging (MRI); positron emission tomography–computed tomography (PET-CT).

The efficient advancement of RT workflows in their entirety relies heavily on integrating imaging, text (reports/guidelines), and structured clinical data [12]. In order to delineate the technical core that enables LLMs to achieve multimodal fusion and clinical workflow integration, Figure 2 presents a multimodal foundation LLM framework for RT applications. Through its hierarchical design, comprising multimodal input, fusion, and clinical application layers, the framework enabled the six core clinical applications of RT to be empowered end-to-end via multisource data integration. It is important to emphasize that multimodal fusion in RT and its integration into clinical workflows involve unique and critical considerations. The extent to which RT is integrated into clinical workflows directly affects the precision with which treatment is delivered [12]. LLMs’ multimodal fusion capabilities precisely addressed the core challenge of integrating multisource data in RT, paving the way for their use in fundamental RT processes such as target delineation and treatment planning.

## 2. Literature Search and Selection

**Figure 3:**
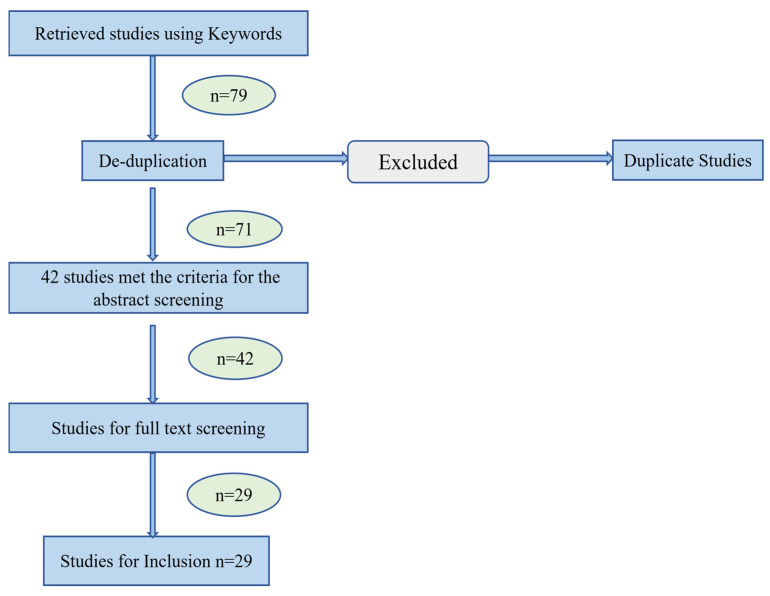
Search and filtering strategy used to select clinical applications of LLMs in RT for inclusion in this review.

We performed a search of the PubMed database for journal articles published in English. We limited our review to studies published over the past 10 years (i.e., the start of 2015 through the end of 2025) to better reflect the contemporary state of LLM-based cancer research. Search keywords included ((“large language models” OR “LLMs”)) AND ((“radiation therapy” OR “radiotherapy” OR “RT” OR “cancer radiotherapy”)). A total of 79 potentially relevant citations were initially identified from the database search. After removing duplicates, 71 records remained. Two authors screened all titles and abstracts for preliminary eligibility. Discrepancies were resolved by consensus or by consulting a third author. The literature inclusion criteria were as follows: (1) original research articles, review articles, and feasibility studies published in English; (2) studies focused on clinical applications of LLMs in RT consistent with the review’s purpose. The exclusion criteria were as follows: (1) meta-analyses, letters, editorials, conference abstracts, case reports, and expert opinions; (2) studies with incomplete data, unclear methodology, or irrelevant outcomes; (3) duplicate publications, protocols; (4) non-English articles without available full translations. Figure 3 describes the scoping review methodology for the article screening and inclusion procedure. Through a thorough review of the remaining literature, we ultimately included 29 studies covering the six core application areas relevant to this review. To enhance the comprehensiveness of the review, subsequent analyses will conduct cross-sectional comparisons of the designs, methodologies, and conclusions of different studies within the same field. This approach aims to reveal the trajectory of technological development, as well as emerging consensuses and divergences.

## 3. Automated Target Volume Contouring

Accurate target volume contouring is essential for RT planning as it directly impacts treatment efficacy and the risk of injury to OARs. LLMs offer a new approach to automated target volume contouring by combining clinical text information with imaging data. Representative multimodal foundation LLMs include Medformer, Radformer and LLMSeg. The following is a deep comparison and critical analysis of its technical route and performance.

The Medformer model employed a hierarchical visual Transformer as its backbone architecture and leveraged LLMs for extracting rich textual features. Through the vision–language attention module, these features were fused with radiological characteristics to guide the segmentation model in identifying target volumes with blurred boundaries [13]. The model was evaluated using an in-house prostate cancer dataset (668 patients) and a public oropharyngeal carcinoma dataset, delivering strong performance in target delineation for both cancer types. For prostate cancer gross target volume (GTV) delineation, the model achieved a Dice similarity coefficient (DSC) of 0.81 ± 0.10 versus 0.72 ± 0.10, an intersection over union (IOU) of 0.73 ± 0.12 versus 0.65 ± 0.09, and a 95th percentile Hausdorff distance (HD95) of 9.86 ± 9.77 mm versus 19.13 ± 12.96 mm [13]. These parameters served as intermediate metrics that may support improved patient prognosis. From a clinical perspective, the higher DSC (0.81 vs. 0.72) reflected greater spatial overlap between the AI-delineated target and the physician’s gold standard, potentially reducing the manual correction and verification workload of physicians. The reduction in HD95 (9.86 mm vs. 19.13 mm) demonstrated the improved boundary and segmentation accuracy of the model, particularly in the precise delineation of tumors with ill-defined margins. The concurrent improvement in IOU further confirmed the model’s enhanced capability in GTV segmentation in exploratory testing.

In oropharyngeal carcinoma GTV delineation, the Medformer model obtained a DSC of 0.77 ± 0.11 and an HD95 of 7.52 ± 4.8 mm, representing significant improvements (*p* < 0.05) [13]. Clinically, a lower HD95 may mitigate the risk of target underdosing or normal tissue overexposure due to GTV boundary misalignment—an important consideration for protecting OARs such as the parotid glands, spinal cord, and brainstem in head and neck cancer RT. For delineating the clinical target volume (CTV), the Medformer model achieved a DSC of 0.91 ± 0.04, an IOU of 0.85 ± 0.05, and an HD95 of 2.98 ± 1.60 mm—a performance on par with other state-of-the-art algorithms [13]. A CTV DSC of 0.91 meant the automated delineations were on par with manual contouring by clinicians. After simple verification, these results may support improved workflow efficiency in exploratory clinical planning. Additionally, the very low HD95 (≈3 mm) may support the accurate setting of safety margins, laying a preliminary technical foundation for precision RT.

Radformer was optimized for the auto-delineation of head and neck cancer target volumes; its overall model design, experimental validation, and clinical conclusions are all from the original research [14]. It adopted two key foundational technologies: the hierarchical Shifted Window (Swin) Transformer [15] as the backbone segmentation architecture, and PubMed Bidirectional Encoder Representations [16] for clinical text feature extraction. The primary function of LLMs within this architecture was feature mining to identify critical information from clinical notes and authoritative literature [16]. On a single-center dataset of 2985 head and neck cancer patients, the model achieved DSC and IOU values of 0.76 and 0.69, respectively, with an HD95 value of 7.82 mm. Compared to 3D-UNETR, Radformer model improved DSC and IOU by 15% and 17%, respectively, and reduced HD95 by 45% [14]. Clinically, the 15% DSC improvement may reduce physician time spent adjusting complex head and neck cancer target volumes (involving multiple anatomical structures), while the 45% reduction in HD95 may enhance boundary recognition accuracy for regions like the larynx and nasopharynx. This avoids redundant safety margins caused by boundary uncertainty, thereby potentially lowering the risk of radiation-induced side effects. This model demonstrated particularly precise boundary identification capabilities in complex target delineation scenarios, such as cases involving laryngeal and nasopharyngeal cancers. Radformer’s core strength is its precise boundary recognition capability for complex head and neck anatomical regions such as the larynx and nasopharynx, which can avoid redundant safety margins caused by boundary uncertainty [14]. However, its major limitation is its over-reliance on a large single-center dataset, and the lack of multi-center data fusion makes it difficult to adapt to the differences in delineation protocols among different institutions; moreover, the model has not been tested in cross-center external validation, so its true clinical efficacy remains to be validated.

LLMSeg is a proof-of-concept multimodal foundation framework that dynamically adjusts contouring strategies by parsing clinical text metadata (tumor stage, surgical procedure, and tumor laterality via LLaMA-7B-chat [17]. For breast cancer CTV contouring, LLMSeg yielded DSC scores of 0.829 and 0.844 on internal and external validation cohorts, respectively, significantly outperforming vision-only segmentation models. Notably, it retained stable performance (DSC > 0.8) with only 40% of the full training dataset, demonstrating data efficiency [17]. However, its application has not been extended to tumors with complex pathological characteristics and diverse surgical procedures (e.g., pancreatic or esophageal cancer), and its performance in the contouring of OARs with irregular boundaries needs further verification.

For cervical cancer (CC) brachytherapy, an LLM-prompted segmentation model extracts contouring rules from clinical practice guidelines (American Society for Radiation Oncology/European society for Therapeutic Radiation Oncology) via ChatGPT to generate task-specific prompting instructions. A retrospective dataset of 32 CC patients, encompassing 124 planning CT images was utilized [18]. This approach enabled accurate segmentation of high-risk clinical target volume (HR-CTV) and OARs, achieving a DSC of 0.795 ± 0.081 for HR-CTV, 0.91 ± 0.07 for the bladder, and an HD95 of 6.324 ± 2.311 mm for HR-CTV [18]. A DSC value of over 0.9 for the bladder may help prevent potential dose limit violations arising from inaccurate OAR delineation, thereby lowering the risk of radiation-induced cystitis. An HD95 of 6.324 ± 2.311 mm for the HR-CTV was critical for enhancing local tumor control rates and reducing late complications in patients with CC. This model innovatively integrates clinical guideline knowledge into the segmentation process, which improves the consistency between automated delineation and clinical practice standards, but its performance for HR-CTV is lower than that of Medformer and Radformer for solid tumor GTV.

In addition to automated contouring, LLM-based models also address the problem of non-standard naming of target volumes and OARs across different medical institutions, which is a key obstacle to multi-center collaborative research [19]. Differences exist in the naming conventions for targets and OARs across medical institutions. GPT-4 (LLM-based) can automatically convert non-standardized anatomical names into standardized terminology based on the American Association of Physicists in Medicine (AAPM) TG-263 guidelines. Its accuracy is comparable to traditional machine learning approaches, whilst requiring no additional training. This capability may facilitate rapid adaptation to multi-center datasets, laying the preliminary foundation for cross-institutional research and data sharing [19].

The comparative analysis of the aforementioned LLMs reveal distinct technical strata and shared challenges in auto-contouring. Medformer/Radformer enhances segmentation accuracy by deeply integrating image and text features. In contrast, LLMSeg dynamically adjusts strategies for analyzing clinical text data through LLaMA-7B-chat, emphasizing data efficiency and clinical knowledge integration. Despite varied technical approaches, all studies are constrained by two common limitations: first, validation is predominantly based on single-center, retrospective data; second, the evaluation indicators only focus on technical surrogate metrics (DSC, HD95, IOU), and there is no correlation analysis with clinical endpoints (e.g., target underdosing rate, OAR radiation injury rate), making it impossible to verify the actual clinical benefit of automated delineation. Meanwhile, the current research also reveals a clear development trend: the integration of clinical text metadata (guidelines, pathological reports, surgical records) and imaging data is the core direction of LLMs in auto-delineation, and the improvement of data efficiency and cross-center generalization ability will be key to the next stage of technical iteration.

## 4. Dose Prediction and Automation of RT Planning

Dose prediction and automated RT planning represent core components for enhancing the efficiency and standardization of RT workflows. Conventional manual planning is time-consuming, labor-intensive, and heavily reliant on the expertise of medical physicists [20,21]. LLMs offer new capabilities to empower dose prediction and treatment planning optimization. The comparative performance of different LLMs in automated RT planning for various tumor types is summarized in Table 1, which clearly reflect their differences in core advantages, process efficiency, and clinical outcomes across cancer types such as lung, cervical, prostate, and head and neck cancers. All findings presented in this section are based on analyses of original peer-reviewed studies [22,23,24,25,26].

### 4.1. DoseGNN Model

Dose prediction serves as a critical prerequisite for optimizing RT planning. Researchers [22] have developed a DoseGNN model that employs LLMs (LLM assisted) to encode treatment prescriptions and physician instructions, thereby predicting dose–volume histograms (DVH) from structures. In dose prediction for intensity-modulated radiotherapy (IMRT) of lung cancer, the model achieved mean absolute error (MAE) reductions of 64%, 53%, 64%, 64%, and 61% for Dmax, Dmean, D95, and D1 within the planned target volume (PTV), respectively, relative to the optimal baseline model [22]. Furthermore, the LLM-enabled DoseGNN model facilitates seamless treatment plan adjustments through natural language interaction with clinicians. A reduction in MAE indicated that predicted doses align more closely with clinically acceptable dose distributions. This minimized the number of plan adjustments required by medical physicists, thereby alleviating the burden of treatment planning. More importantly, precise dose prediction may mitigate the potential risks of target volume underdosing and overexposure of OARs. This is critical for balancing therapeutic efficacy and treatment safety in RT. However, the study only reports the improvement of dose prediction accuracy at the technical level and does not further validate whether the accurate dose prediction can reduce the number of manual plan adjustments in actual clinical work, leading to the disconnection between technical performance and clinical efficiency improvement.

### 4.2. Multi-Agent Collaborative Automated Planning System

GPT-Plan (multimodal system) is a pioneering multi-agent collaborative system for automated RT planning optimization, inspired by LLMs’ strong knowledge reserve and reasoning ability. Its system design, experimental validation, and clinical conclusions were derived from the original research [23]. This system comprises four core agents: the Dosimetrist Agent generates and optimizes objective function weights, dose constraints, and other optimization parameters (OPs); the Treatment Planning System (TPS). Proxy Agent interacts with the TPS to generate dose distributions based on OPs; the Physicist Agent evaluates dose outcomes against clinical standards and provides improvement feedback; and the Human proxy Agent supports optional human intervention, balancing automation with flexibility [23]. GPT-Plan also features auxiliary tools and mechanisms to mitigate LLM hallucinations, utilize historical plans, and balance exploration and utilization, with a modular design that enables flexible adaptation to various clinical scenarios [23].

In 12 cases of IMRT for lung cancer, the GPT-Plan generated improvements in technical metrics for target coverage (D95 increased by 4.75%, *p* < 0.05) and dose uniformity, as indicated by a 49.52% reduction in the homogeneity index (HI, *p* < 0.05). The mean dose to healthy lung tissue was decreased by 14.31% (*p* < 0.05), and the volume receiving 5Gy (V5) was reduced by 17.60% (*p* < 0.05), accompanied by improved spinal cord sparing [23]. For CC, its therapeutic effect was comparable to that of senior physicists, and its OAR protection performance was consistently superior to that of junior physicists (rectal V50 reduced by 10.34%, bladder mean dose reduced by 17.47%, bowel V40 and mean dose were lowered by 15.80% and 14.67%, and both V20 and mean dose to the femoral heads were significantly reduced) [23]. These results indicate that GPT-Plan can standardize RT planning quality and reduce the impact of planner experience variation on treatment efficacy. The reduced doses to OARS lower the incidence of late complications such as radiation proctitis, cystitis, and femoral head necrosis, thereby improving patients’ quality of life after treatment.

The clinical safety design of GPT-Plan permeates the entire multi-agent collaborative workflow, with three core safeguard mechanisms integrated as follows. Clinical constraints: Based on RTOG guidelines, the system sets organ priorities and dose limits. The Dosimetrist Agent generates OPs, and the TPS Agent monitors compliance, forming a dual-constraint system. The Physicist Agent further validates clinical suitability. Two-stage fault detection: Basic errors are first identified; then, complex logical faults are detected by Mirror LLM and corrected within 2–3 iterations. Mandatory human oversight and final approval: Mandatory manual review is activated in high-risk situations, and all plans require final expert approval to ensure clinical safety.

### 4.3. Multimodal Fusion Automated Planning Framework

GPT-RadPlan was built upon the GPT-4V multimodal foundation LLM and was the first agent of its kind to simulate human planner behavior in clinical radiation oncology. Leveraging contextual learning, GPT-RadPlan integrated optimized settings and clinical requirements from three approved clinical plans. For specific patients, GPT-RadPlan assumed dual roles as both plan evaluator and planner. It first assessed DVHs to determine their alignment with clinical standards, generating textual feedback. Subsequently, it adjusted optimization parameters (e.g., weights, dose targets) based on this feedback, iteratively refining the plan. This system is integrated with internal inverse treatment planning systems, requiring no domain-specific training. Learning clinical decision logic from a small number of reference plans, it supports adjustments via natural language interaction and adapts to treatment needs across different cancer types [24]. A study by Wang Q also investigated the application of comparable agent-based frameworks in automated treatment planning, further validating the increasing value and potential of LLM-driven techniques in clinical RT [23].

This automated planning system [24] was validated using VMAT plans in 17 prostate cancer cases (prescribed dose: 70.2 Gy) and 13 head and neck cancer cases (prescribed dose: 72 Gy) by comparing the performance of GPT-RadPlan with those of clinical plans devised by human specialists. Results demonstrated that GPT-RadPlan outperformed or equaled clinical plans in all cases, exhibiting superior target coverage while reducing OAR doses by an average of 5 Gy (15% reduction in prostate cancer; 10–15% reduction in head and neck cancer). Moreover, the number of iterations (3–6) was comparable to that of human planners and was lower than AutoPlan (7–10 iterations) and the Bayesian optimization (BO) baseline (over 50 iterations) [24]. Fewer iteration cycles shorten planning duration, improve clinical workflow efficiency, and allow for earlier treatment initiation, which is especially advantageous for patients with rapidly progressive tumors [25].

In CC RT, researchers [26] employed three LLMs, namely Qwen-2.5-Max (Alibaba Cloud, Hangzhou, China), Gemini-1.5-Flash (Google, Mountain View, CA, USA), and Llama-3.2 (Meta AI, Menlo Park, CA, USA), adapted to clinical requirements through prompt engineering. The feasibility of automated planning was validated using data from 35 CC patients (customized prompts were applied to 5 patients to tailor the LLMs, which were subsequently tested on 30 patients). Research findings indicated that Gemini-1.5-Flash generated hallucinations, leading to confusion in OAR dose assessment and rendering it incapable of producing clinically acceptable plans, whereas Qwen-2.5-Max and Llama-3.2 demonstrated exceptional performance, generating clinically compliant VMAT plans within 16.3 ± 5.0 min and 9.8 ± 2.1 min, respectively [26]. This processing time was shorter than that required for manual planning by senior physicists (approximately 20 min). All patients’ Llama-3.2 iterations required fewer than 11 iterations, while Qwen-2.5-Max required fewer than 18 iterations, indicating the method’s adaptability to diverse target volumes and OARs locations. Patient-specific quality assurance results demonstrated the gamma pass rate for LLMs plans exceeded 0.995 (2 mm/2%), which was higher than that of manually created plans (0.99), thus verifying the clinical feasibility of plan delivery [26]. The high pass rate reduces the need for repeated dose verification and plan adjustments, thereby decreasing the quality control burden on physicists.

### 4.4. Efficiency Analysis in Automated Planning/Hallucinations Mitigation

Efficiency improvement and hallucination mitigation are the key to the clinical application of LLM-driven automated RT planning systems. In terms of efficiency, GPT-Plan (with Retriever) required only 2–4 iterations (average 3.2) to obtain the optimal CC plan, which was comparable to senior physicists (3–6 iterations, average 4.0); the Wilcoxon signed-rank test revealed no statistically significant difference (W = 11, *p* = 0.313) [23]. GPT-RadPlan completed plan optimization in 3–6 iterations, which was fewer than AutoPlan (7–10 iterations) and the BO baseline scheme (more than 50 iterations) [24]. The retrieval tool accelerates the convergence process of the model, stabilizes the optimization workflow, and makes the optimization trajectory smoother with fewer sudden changes [23]. GPT-RadPlan can be integrated with Retrieval-Augmented Generation (RAG) to retrieve the most similar plans from the database for assistance [24].

LLM hallucination represents the primary risk factor compromising the accuracy of automated treatment planning, and targeted mitigation strategies with favorable clinical performance have been developed in a recent study [27]. GPT-Plan employed a two-stage self-reflection framework [27]: in the first stage, an automated quality control module verified treatment plan formatting, structure naming conventions, and parameter combinations, achieving a 100% detection and correction rate for nonconforming items; in the second stage, an independent mirrored LLM agent assessed the rationality of the planning strategy and its consistency with optimization objectives, with logical hallucinations effectively corrected within 2–3 iterative cycles [27]. Similarly, Qwen-2.5-Max and Llama-3.2 mitigate hallucinations and ineffective optimization adjustments through structured prompted that explicitly define clinical criteria and dose constraints [26].

As summarized in Table 1, a comparative analysis of different LLMs for automated planning reveals clear evolutionary trends. Architectural Shift to Autonomous Workflows: GPT-Plan [23] and GPT-RadPlan [24] signify a paradigm shift, enabling end-to-end autonomous planning. This evolution aligns more closely with the holistic clinical workflow of RT physicists. The inherent trade-off between safety and efficiency is that high-performance systems navigate the safety–efficiency balance differently. GPT-Plan ensures high safety through a sophisticated multi-agent design with multi-layered verification, which comes with inherent architectural complexity. GPT-RadPlan’s single-agent structure balances safety and efficiency; RAG is pivotal in accelerating convergence, improving output stability, and enhancing generalization. Clinical institutions should select models based on their technical conditions and clinical needs, which is a critical principle for the clinical transformation of LLM-driven automated planning technology. The complete failure of Gemini-1.5-Flash [26] to produce clinically acceptable plans due to severe hallucinations provides a critical warning. It underscores that inherent model reliability and robustness are non-negotiable prerequisites for any clinical application.

Nevertheless, this field faces some limitations. First, evaluation indicators focus on technical metrics and ignore clinical workflow efficiency, although an existing study [26] measures the actual time for physicists. Second, the models are not integrated with RT quality control and follow-up systems, so they are isolated tools in the RT workflow and cannot form a closed loop of “planning–quality control–treatment delivery–follow-up,” making it difficult to maximize their clinical value. Additionally, the field has common limitations such as small-sample retrospective validation and model hallucinations, which are analyzed in Section 8.

## 5. Patient Education and Communication

Patients receiving RT often face difficulties in understanding complex medical terms and treatment processes, and insufficient access to accurate information is likely to cause anxiety and reduce treatment compliance. LLMs have been fully validated in the education of adult patients with lung cancer and meningioma, and the core conclusion is that they have good information expression and relevance, but there are deficiencies in content completeness, with conclusions derived from [28,29].

Six multidisciplinary clinicians specializing in lung cancer treatment (including radiation oncologists, medical oncologists, and thoracic surgeons) evaluated the responses from ChatGPT-4o (July 2024 version) to eight common categories of questions regarding lung cancer RT education. These categories covered disease understanding, treatment plans, and side effect management. The clinicians assessed the responses’ relevance, accuracy, and completeness using a five-point Likert scale. Results indicated that clinicians awarded high scores for relevance (4.5 points) and accuracy (4.3 points), while patients also provided positive feedback on information clarity (4.4 points) and relevance (4.3 points). However, credibility (mean 3.8; standard deviation (SD): 0.68) and practicality (mean: 3.7; SD: 0.73) scored relatively lower. No harmful misinformation was identified in responses. The scoring parameters are intermediate indicators that may indirectly improve prognosis by enhancing patients’ information acquisition efficiency and treatment adherence. The results show that the supervision of clinicians is still necessary. Consequently, clinician supervision remains necessary, and further optimization of LLM-generated content is required. ChatGPT-4 holds promise as a supplementary tool for radiation oncology patient education [30].

Diana Coralia Dehelean et al. [28] posed eight questions to ChatGPT 4 concerning meningioma diagnosis, treatment plans, and RT. This study employed a dual-perspective approach involving both patients and clinicians, with seven uninformed clinicians scoring each model’s responses to identify the most accurate, persuasive, and comprehensive answers overall. In the context of meningioma patient education, the majority perceived the information provided by ChatGPT as clear, accurate, and consistent with their personal experiences. Sixty percent of patients expressed a willingness to use ChatGPT for future medical consultations. Clinicians awarded high scores for the relevance and accuracy of the information, although completeness scores were slightly lower, particularly for specific RT details and questions concerning treatment-related side effects. Overall, Diana Coralia Dehelean concluded that ChatGPT holds significant value as a patient education tool.

Current research primarily focuses on applying GPT models to adult RT. In pediatric RT, patients and their guardians usually only meet the radiation oncologist in the later stages of treatment. During this period, they often search online for information about RT, but the reliability of these sources is often questionable. Dominik Wawrzuta and colleagues collected questions about radiation oncology from pediatric RT patients aged 10 years and older, as well as from their parents [29]. The team then compared the responses provided by GPT-3.5, GPT-4 and a fine-tuned GPT-3.5 model with those of an experienced pediatric radiation oncologist. The responses were evaluated across three dimensions: reliability, conciseness, and comprehensibility. The results suggested LLMs could be valuable tools for educating patients and their families before treatment in pediatric radiation oncology. Of these models, only GPT-4 produced responses of a quality comparable to that of radiation oncologists, although it occasionally provided inadequate answers. GPT-3.5 models should be used cautiously as they are more likely to produce inadequate answers to patient questions [29]. This population-specific performance difference indicates that LLM-driven patient education needs to select models and customize prompts according to the characteristics of different populations, and a one-size-fits-all application is not feasible.

In summary, the version of LLM is the core determinant of performance in patient education: models like GPT-4/4o can provide high-quality information, though content completeness requires further improvement. In higher-risk, personalized decision-support scenarios (e.g., pediatric oncology or complex case counseling), lower-capability models like GPT-3.5 may generate inadequate responses. Most importantly, physician supervision is a mandatory prerequisite for clinical application: no LLM can completely replace physicians to carry out RT patient education, and all studies emphasize the necessity of physician supervision and review of LLM-generated information to avoid the potential risk of misinformation and ensure that the information is consistent with the patient’s individual clinical situation.

## 6. Clinical Decision Support

The scientific rigor and accuracy of clinical decision making in RT directly affects the applicability of treatment plans and the long-term prognosis of patients. Current LLMs in this field are all in the proof-of-concept stage with certain application potential and limitations. Specific applications and achievements are as follows:

In head and neck cancer clinical trial recommendation, a retrieval-augmented GPT-4 combined with the Memorial Sloan Kettering Cancer Center’s LookUpTrials real-time database achieved a precision of 63.0%, recall of 100.0% and F1-score of 0.77 in matching physician’s clinical trial recommendations [31], which can effectively assisted clinicians in screening appropriate trial programs for patients and improve the efficiency of clinical trial enrollment. These scoring parameters were intermediate indicators that could indirectly support improved patient information acquisition efficiency and treatment adherence in theoretical scenarios. The results showed that the supervision of clinicians is still necessary. In terms of treatment planning, the MEREDITH system-built upon Google Gemini Pro- innovatively combined RAG with chain-of-thought reasoning. It incorporated PubMed-indexed literature, clinical trial databases (e.g., ClinicalTrials.gov), drug approval statuses and multiple editions of oncology guidelines (e.g., American Society for Medical Oncology and European Society for Medical Oncology). Treatment plans generated for 10 publicly available fictional tumor cases achieved a semantic similarity of 0.76, with recommendations from Molecular Tumor Board (MTB) experts, representing a significant improvement (*p* = 0.01) over the initial version (0.71) [32]. This framework offers reliable intelligent decision support for clinical cancer treatment planning.

During the diagnosis and treatment of specific cancer types, GPT-4o attained the highest area under the curve (AUC = 0.848) for breast cancer tumor classification during treatment planning based on patients’ clinical histories, pathological characteristics, and genomic profiles [33]. As an intermediate indicator, this performance provides supportive evidence for precision oncology in proof-of-concept testing. Furthermore, physician evaluations indicated that DeepSeek-R1 received the highest ratings for disease interpretation (4.73 ± 0.46) and treatment recommendations (4.70 ± 0.51), with consistent scoring outcomes, which provided multiple choices for the individualized diagnosis and treatment of breast cancer [33].

In soft tissue sarcoma diagnosis and management, Claude 3.5 Sonnet achieved 60% concordance with recommendations from the Multinational Sarcoma Board across 21 sarcoma centers. However, all LLMs exhibited recommendation biases, and some models even proposed harmful and guideline-inconsistent suggestions (e.g., unnecessary amputation) [34], indicating the need for strict clinical verification of LLM-generated treatment recommendations.

Beyond core tumor diagnosis and treatment decisions, researchers had developed a domain-specific chatbot, RTPhy-ChatBot, tailored for RT physics design. Its knowledge base comprised AAPM reports, utilizing the Meta-LLaMA3-8B-Instruct model to construct a RAG Querying framework. This design enabled the chatbot to provide fact-based responses grounded in authoritative publications, while safeguarding privacy and ensuring traceability. Testing with 20 template questions revealed that RTPhy-ChatBot achieved an average score of 4.0 ± 0.9 across these queries, outperforming Gemini-2.0-Flash (3.9 ± 1.1), GPT-4o (4.0 ± 1.4), and Moonshot-v1 (3.8 ± 1.2). Rouge analysis as an intermediate indicator can enhance the efficiency and accuracy of physical planning design in exploratory research. Rouge analysis revealed Rouge-1 scores of 0.5127, Rouge-2 of 0.2119, and Rouge-L of 0.2748, comparable to commercial LLMs [35]. The result demonstrated exceptional performance on questions involving specific quality assurance standards.

The RTPhy-ChatBot customized with AAPM reports outperforms mainstream commercial LLMs, indicating that the performance of general LLMs in RT-specific decision tasks is limited, and domain-specific fine-tuning using RT guidelines/databases and integration with RAG technology are the keys to improving performance. In addition, the application of LLM in RT field also has common limitations such as single-center small-sample validation, and the hallucination problem is more prominent in complex decision tasks, which needs to be solved by systematic technical optimization and clinical governance (see Section 8).

## 7. Information Extraction and Tumor Prognosis Application

### 7.1. Information Extraction

LLMs have strong natural language processing and unstructured data mining capabilities, which break through the limitations of traditional methods in extracting clinical information from pathological reports, radiological reports, and medical literature.

Regarding pathology report extraction, GPT-3.5-turbo achieved an overall accuracy of 87.7% when processing 340 breast cancer patient reports for key indicators including tumor size, lymph node metastasis status, histological grade, molecular subtype, and lymphatic vessel invasion [36]. Lymphatic vessel invasion extraction reached an accuracy rate of 98.2%, and large-scale processing was completed within 4 h [36]. In radiological report information extraction, GPT-4 demonstrated superior performance to ChatGPT in extracting lesion parameters from lung cancer CT reports, achieving accuracy rates of 98.6% versus 84.0% (*p* < 0.001) and a correct report extraction rate of 96% (compared to ChatGPT’s 67%, *p* < 0.001). GPT-4 demonstrated greater accuracy in identifying metastatic disease (98.1% [95% CI: 97.7, 98.5] versus 90.3%) [37].

ChatGPT achieved a 90% accuracy rate in evaluating literature on proton therapy for lung cancer, demonstrating 3229 times greater efficiency than manual assessment [38]. The model also enabled rapid extraction of imaging features associated with complications including radiation esophagitis and pneumonitis [38]. The effectiveness of different LLMs in extracting information from pathological reports, radiological reports, and related studies is summarized in Table 2, demonstrating their high accuracy and efficiency in processing unstructured clinical text data.

Synthetic comparative analysis indicates that all included LLMs achieve high accuracy in standardized, clear text extraction tasks such as pathological indicators and radiological lesion parameters. Nevertheless, performance varies sharply by model architecture: GPT-4 outperforms GPT-3.5-turbo in complex semantic and diagnostic extraction, while simpler models remain sufficient for structured, low-complexity tasks. All current studies are limited to retrospective, single-source, single-tumor validation, lacking multi-center, multi-disease, and real-world prospective testing. Therefore, although LLMs demonstrate promising efficiency and accuracy in radiation oncology information extraction, their universal clinical applicability and stability require further verification.

### 7.2. Prognosis Assessment

LLMs integrate extracted clinical information with prognostic correlates to construct efficient predictive models, offering an innovative pathway for evaluating treatment tolerance, complication risks, and long-term survival in cancer patients. In RT complication prediction, researchers combined LLMs with meta-analysis to build a prediction model for lung cancer proton therapy complications [37]. The model achieved an effect size of 0.79 for predicting radiation esophagitis and 0.77 for radiation pneumonitis, with a HI of 0% for both indicators [38], indicating high consistency and reliability in predicting specific RT complications, which can effectively assist clinicians in formulating individualized intervention plans for high-risk patients.

In real-time symptom monitoring and prognosis improvement of head and neck cancer patients, LLMs showed direct clinical benefits [39]. An LLM-RAG based electronic patient-reported outcome (ePRO) system was developed for real-time monitoring of symptoms in head and neck cancer patients receiving RT [39]. The results showed that high-frequency use of the system (≥6 submissions during treatment) significantly reduced the patient’s weight loss rate and treatment interruption days, improved the effect of clinical symptom control, and thus indirectly improved the overall treatment prognosis of patients [39]; this system realizes the real-time interaction between patients and medical staff, and makes the clinical intervention more targeted and timely [39].

To summarize, LLMs can effectively assist in the prediction of RT complications and real-time monitoring of patient symptoms, providing a novel and efficient approach for tumor prognosis assessment in radiation oncology. However, the current prognosis prediction models are mostly based on retrospective data, and their prospective prediction ability and external generalization still need further clinical verification.

## 8. Limitations and Future Research Paths

The previous discussion has delineated the promising role of LLMs in optimizing clinical efficiency and standardizing practice in six core domains of radiation therapy. Nevertheless, the application of LLMs in RT remains in the early exploratory phase, with multiple inter-related limitations that jointly hinder their large-scale clinical deployment. These limitations can be analyzed into four critical dimensions, namely, study design, technical performance, evaluation systems, and clinical implementation, all of which directly impact the safety, reliability, and generalizability of LLMs in RT clinical practice.

1. Restrictive study design undermines clinical extrapolation: Most foundational studies on LLMs in RT adopt small-sample, single-center, retrospective designs, introducing inherent selection and center-specific biases. Limited sample sizes (e.g., 35 patients for LLM-based automated CC RT planning [26]; 668 single-center prostate cancer cases for Medformer validation [13]) fail to capture the clinical diversity of tumor heterogeneity, patient variability, and equipment differences in real-world RT practice, leading to poor performance reproducibility. Additionally, models trained on single-center data (e.g., Radformer, developed on 2985 single-center head and neck cancer patients [16]) are prone to overfitting to local clinical workflows, delineation standards, and dose-planning preferences—factors that vary widely across institutions. GPT-Plan and GPT-RadPlan [23,24] lack large-scale prospective and multicenter validation, precluding verification of their long-term stability and allowing for overestimation of performance due to retrospective data biases. This represents the primary barrier to translating LLM research into cross-center clinical practice.
2. Core technical bottlenecks pose clinical safety risks: “Hallucination” is the most critical technical risk for LLM application in RT. For instance, Gemini-1.5-Flash produced clinically infeasible CC RT plans due to hallucinations [26], which could lead to severe clinical consequences such as target underdosing or excessive normal tissue irradiation. These limitations have been rigorously reported in high-quality clinical studies [40,41]. In clinical decision support, some LLMs proposed guideline-inconsistent and potentially harmful recommendations, such as unnecessary amputation for sarcoma patients [34]. These cases demonstrate that “hallucination” is not a theoretical concern but a technical flaw with direct potential for clinical harm. Furthermore, most LLM-RT models are customized for single tumor types or isolated clinical tasks, with poor generalizability to complex scenarios (concurrent multi-cancer RT, rare tumor treatment, special populations) due to insufficient representative training data. These limitations introduce uncontrollable safety risks, failing to meet the high reliability requirements for medical AI in RT.
3. Heterogeneous evaluation systems impede comparative validation and translation: A standardized, clinical-oriented evaluation system for LLMs in RT is currently lacking. Existing studies exhibit significant heterogeneity in indicator selection: target delineation research either uses a single metric (DSC [17]) or a composite panel (DSC, IOU, HD95 [14]), while automated planning research prioritizes either DVH optimization [23] or gamma pass rates [26]. This heterogeneity precludes direct cross-study comparison of model performance. More importantly, evaluations rely heavily on technical surrogate indicators (e.g., DSC, MAE, gamma pass rates) with no established correlation to core RT clinical endpoints (e.g., treatment-related complication rates, local tumor control, long-term patient survival) [42]. The disconnect between technical optimization and clinical benefit limits the scientific validation of LLM clinical value and creates a barrier for regulatory approval and clinical translation.
4. Non-technical barriers create systemic obstacles to clinical implementation: Even with technical/design improvements, LLM translation faces unresolved non-technical challenges in medical liability, regulation, data governance, and clinical governance. LLM-RT systems lack unified regulatory classification, approval, and access standards, as existing medical device frameworks fail to adapt to the iterative and cross-modal characteristics of LLMs. Previous studies have proposed the establishment of a standardized clinical governance system incorporating hierarchical review, strict quality control, and mandatory human verification [43,44]. It is also essential to clarify that clinicians bear the ultimate legal responsibility [43,44,45] and to strictly comply with regulatory requirements concerning the approval and classification of AI-powered medical devices [33]. RT clinical data (imaging, text, pathology) contain sensitive patient information, yet LLM training requires large-scale multicenter data sharing—a conflict unresolved by the absence of RT-specific standardized datasets and privacy-preserving computing frameworks. Finally, no full-lifecycle clinical governance system exists for LLM-based RT tools, including standardized operating procedures, mandatory manual verification nodes, or long-term performance monitoring, compromising the stability and safety of clinical applications.

These limitations are interdependent: restrictive study designs limit real-world model validation; technical bottlenecks introduce clinical risks; heterogeneous evaluation systems prevent objective performance assessment; and non-technical barriers block the translational pathway from research to practice. Notably, these are not inherent flaws of LLM technology but rather growing pains of early-stage research in RT, which also define the core priorities for future development.

In the future, the specific applications of LLMs in the field of RT can be categorized into the following dimensions:

1. Technical optimization and performance enhancement: Address the “hallucination” issue through refined RAG and multi-stage self-reflection mechanisms to improve output accuracy, and overcome limitations of single-data-type models to enhance generalizability in complex clinical scenarios (e.g., multi-cancer concurrent RT, rare tumor treatments).
2. Deepening clinical application scenarios: Explore LLM applications in RT quality control and multi-center collaborative quality assurance studies. Standardized workflows and intelligent verification will enhance treatment consistency.
3. Data security and ethical framework development: Establish specialized datasets and annotation standards for RT, balancing data sharing with privacy protection. Formulate ethical guidelines and access criteria for clinical LLM applications to ensure compliance with medical safety regulations.
4. Cross-disciplinary collaborative innovation: Strengthen interdisciplinary cooperation between LLMs and RT physics, clinical medicine, and computer science to develop specialized niche models (e.g., pediatric RT education models). Address complex challenges such as dynamic dose optimization and adaptive RT, providing robust technological support for precision, efficiency, and personalization in RT.

Overall, LLMs represent a revolutionary technological advancement for RT. While significant challenges remain, ongoing technological iteration and deepening clinical validation will inevitably elevate their role in enhancing treatment quality, reducing healthcare burdens, and improving patient outcomes. This progress will drive radiation oncology toward a new era of intelligent practice.

## 9. Conclusions

This narrative review synthesizes the current landscape of LLM applications across the RT workflow. The key finding is that LLMs, by virtue of their superior natural language processing and cross-modal fusion capabilities [8,12], provide a novel auxiliary technical approach to address long-standing challenges in RT, including inter-observer variability in target delineation, inefficient manual planning, and multisource clinical data integration [13,23]. In six core domains (automated target volume contouring, dose prediction and planning automation, patient education, clinical decision support, medical information extraction, and prognosis assessment) [13,23,33,37], Medformer, Radformer and GPT-Plan have demonstrated preliminary improvements in operational accuracy and workflow efficiency for lung, cervical, prostate, and head and neck cancer RT in single-center settings, showing potential to promote the standardization of RT clinical pathways [23,26].

However, a critical synthesis of the evidence reveals a pronounced gap between this potential and the current state of validation. Nearly all existing evidence for LLM applications in RT is derived from retrospective, proof-of-concept, and small-sample single-center studies [26,42], with no large-scale multicenter prospective validation available to date. Although partial models have achieved acceptable technical metrics (e.g., DSC for target delineation, gamma pass rate for planning) [13,26], these intermediate indicators have not been linked to clinically meaningful endpoints such as treatment complication rates or long-term patient survival [42], and inconsistent evaluation metrics across studies [14,17] preclude objective cross-study comparison of LLM performance.

The clinical translation of LLMs in RT is further hindered by multiple under addressed limitations [42,43]. Technically, inherent model hallucinations and poor generalizability [26] pose potential clinical risks (e.g., erroneous OAR dose assessment), and over-reliance on single-center data limits external validity [13,16]. The lack of unified, clinically relevant evaluation benchmarks [42] impedes objective performance assessment and technological iteration. Non-technically, unresolved issues including clinical data privacy protection, ambiguous medical liability for AI-assisted decision making, and incomplete regulatory and ethical governance frameworks [43,44,45] remain unaddressed core prerequisites for safe clinical adoption.

Therefore, the clinical translation of LLMs in RT is not merely a matter of technical refinement but a systemic challenge requiring coordinated progress on three fronts: First, technical strategies (e.g., RAG, multi-stage self-reflection [27]) should be optimized to mitigate hallucinations and enhance LLMs’ generalizability for complex clinical scenarios. Second, a unified evaluation benchmark system [42] should be established, and large-scale multicenter prospective studies should be conducted to verify the correlation between LLM technical performance and clinically meaningful endpoints. Third, standardized clinical governance protocols and improve ethical and regulatory frameworks for data privacy and medical liability should be developed [44,45]. Only through the synergistic advancement of technology, rigorous clinical evaluation, and sound governance can LLM research transition from promising pilot studies to safe, effective, and scalable components of intelligent radiotherapy practice.

## Figures and Tables

**Table 1:** Comparative performance of different LLMs in automated RT planning for various tumor types.

Research Type	Dataset Size	Category of Model	Ground Truth	Core Advantage	Process Time	Iterations
retrospective	12 lung cancer IMRT cases and 5 CC VMAT cases	GPT-Plan [23]	D95 increased by 4.75%, HI decreased by 49.52%, and mean lung dose decreased by 14.31% (*p* < 0.05) The efficacy of the GPT-Plan was comparable to that of senior physicists; OAR protection was superior to that achieved by junior physicists in CC cases.	A pioneering multi-agent system for automated RT planning optimization	-	-
retrospective	5 CC VMAT cases	GPT-Plan (with Retriever) [22]	The efficiency of the system is comparable to that of senior human planners.	Simultaneously enhance the stability and efficiency of optimization.	-	2–4 (average 3.2)
retrospective	17 prostate cancer VMAT cases and 13 head and neck cancer VMAT cases	GPT-RadPlan based on GPT-4V [24]	Reducing OAR doses by an average of 5 Gy (15% reduction in prostate cancer, 10–15% reduction in head and neck cancer)	No additional training required, with strong adaptability and excellent target coverage. Superior OAR protection.	-	3–6
retrospective	35 cases of CC (VMAT)	Qwen-2.5-Max (Alibaba Cloud, Hangzhou, China) [26]	The gamma pass rate > 0.995 (2 mm/2%)	Rapid generation of an acceptable clinical plan	16.3 ± 5.0 min	<18
	35 cases of CC (VMAT)	Gemini-1.5-Flash (Google, Mountain View, CA, USA) [26]	Gemini-1.5-Flash generated hallucinations, leading to confusion in OAR dose	Cannot generate a clinically acceptable plan	-	-
	35 cases of CC (VMAT)	Llama-3.2 (Meta AI, Menlo Park, CA, USA) [26]	The gamma pass rate > 0.995 (2 mm/2%)	Rapid generation of an acceptable clinical plan	9.8 ± 2.1 min	<11

**Table 2:** Comparative effectiveness of different LLMs in radiation oncology information extraction settings.

Research Type	Extract Scene	Dataset Size	Category of Model	Core Conclusions
Retrospective	Pathological report [36]	340 breast cancer patients	GPT-3.5-turbo	The overall accuracy rate was 87.7%, and the lymphatic vessel infiltration extraction accuracy rate was 98.2%.
Retrospective	Radiation report [37]	3523 Lung Cancer CT Reports	GPT-4/ChatGPT	The GPT-4 model outperforms ChatGPT; GPT-4 achieves 98.6% accuracy in lesion parameter extraction and 96% correct reporting extraction rate.
Retrospective	Studies [38]	1569 articles	ChatGPT	Evaluation efficiency is 3229 times higher than manual assessment.

## Data Availability

No new data were created or analyzed in this study.

## Abbreviations

LLMs	Large language models
RT	Radiotherapy
OARs	organs at risk
LD	Linear dichroism
PTV	planned target volume
DSC	Dice similarity coefficient
IOU	intersection over union
HD95	Hausdorff distance 95
SWIN	shifted window
GPT-4	Generative Pretrained Transformer 4
AAPM	American Association of Physicists in Medicine
IMRT	intensity-modulated radiotherapy
VMAT	Volumetric Modulated Arc Therapy
MAE	the mean absolute error
Dmax	the maximum dose
Dmean	mean dose
D95	95% volume dose
D1	1% volume dose
Ops	optimisation parameters
TPS	Treatment Planning System
SD	standard deviation
RAG	Retrieval-Augmented Generation
MTB	Molecular Tumor Board
HR-CTV	High-risk clinical target volume
CC	cervical cancer
CTV	clinical target volume
GTV	Gross target volume
DVH	Dose volume Histogram
HI	Homogeneity Index
ePRO	Electronic patient-reported outcome
AI	artificial intelligence
RTOG	Radiation Therapy Oncology Group
CT	Computed tomography
MRI	Magnetic resonance imaging
PET-CT	Positron emission tomography–computed tomography
BO	Bayesian optimization
AUC	area under the curve
MTB	Multinational Sarcoma Board
